# Elevated VEGF levels contribute to the pathogenesis of osteoarthritis

**DOI:** 10.1186/1471-2474-15-437

**Published:** 2014-12-17

**Authors:** Quan Yuan, Li Sun, Jian-Jun Li, Chun-Hou An

**Affiliations:** Department of Orthopedics, Shengjing Hospital of China Medical University, Sanhao Street No. 36, Heping District, Shenyang 110004 P.R. China; Department of Nephrology, The First Affiliated Hospital of China Medical University, Shenyang, 110001 P.R. China

**Keywords:** Vascular endothelial growth factor, Osteoarthritis, Pathogenesis, Meta-analysis

## Abstract

**Background:**

The aim of our meta-analysis is to understand the relationship between the pathogenesis of osteoarthritis and the expression levels of vascular endothelial growth factor (VEGF) in multiple disease tissues in osteoarthritis patients.

**Methods:**

The following electronic databases were searched, without language restrictions, to retrieve published studies relevant to VEGF and osteoarthritis: MEDLINE (1966 ~ 2013), the Cochrane Library Database (Issue 12, 2013), EMBASE (1980 ~ 2013), CINAHL (1982 ~ 2013), Web of Science (1945 ~ 2013) and the Chinese Biomedical Database (CBM) (1982 ~ 2013). Meta-analysis of the extracted data was performed using the STATA statistical software. Standardized mean difference (SMD) with its corresponding 95% confidence interval (95% CI) was calculated.

**Results:**

A total of 11 case–control studies, containing 302 osteoarthritis patients and 195 healthy controls, met our selection criteria for this meta-analysis. Our analyses of the data available from multiple disease tissues demonstrate that VEGF expression levels in osteoarthritis patients are significantly higher than healthy controls (SMD = 1.18, 95% CI: 4.91 ~ 9.11, *P* < 0.001). A subgroup analysis based on ethnicity revealed that both Asian and Caucasian osteoarthritis patients had higher levels of VEGF expression compared to their respective healthy counterparts (Asians: SMD = 5.49, 95% CI: 3.44 ~ 7.54, *P* < 0.001; Caucasians: SMD = 15.17, 95% CI: 5.21 ~ 25.13, *P* = 0.003; respectively). We also performed other subgroup analyses based on country, language and sample source, and the results showed that, in all these subgroups, osteoarthritis patients had higher levels of VEGF expression than healthy controls (all *P* > 0.05).

**Conclusion:**

Our meta-analysis provides evidence that higher VEGF expression levels strongly correlate with the pathogenesis of osteoarthritis.

**Electronic supplementary material:**

The online version of this article (doi:10.1186/1471-2474-15-437) contains supplementary material, which is available to authorized users.

## Background

Osteoarthritis is a common age-related degenerative musculoskeletal disease, which is associated with declining joint functions, muscle volume and power, with serious impact on daily activities and quality of life[1, 2]. In osteoarthritis, a breakdown of the extracellular matrix (ECM) of articular cartilage occurs in the affected joints, and the disease pathogenesis involves cartilage destruction, bone remodeling and inflammation of the synovial membrane. Osteoarthritis is the most prevalent of chronic rheumatic diseases worldwide, affecting approximately 40% of people over 70 years in age, and is the fourth leading cause of disability[3–5]. As the world population is aging, the incidence and prevalence of osteoarthritis is rapidly increasing and is placing an enormous burden on the health care systems across the world[6]. Epidemiological studies have identified various risk factors in the pathogenesis of osteoarthritis, including age, sex, race, and obesity[7, 8]. Importantly, molecular mechanisms involved in osteoarthritis progression have been the subject of interest for discovery of new pathways and for development of novel strategies for disease modification[9, 10]. In this context, the vasculature has an essential role in osteogenesis, skeletal development and bone fracture repair[9], and in osteoarthritis, disturbances in blood flow due to altered vasculature can have devastating effects on cell survival and fluid drainage.

Vascular endothelial growth factor (VEGF) is a potent angiogenic factor and a critical regulator of angiogenesis in skeletal development and bone remodeling, and VEGF is suspected to play an active role in the pathogenesis of osteoarthritis[11]. VEGF functions as a homodimer and is a highly glycosylated protein of 46–48 kDa, with strong angiogenic activity, and elicits mitogenic and chemotactic responses in endothelial cells[12]. VEGF is considered to be a potent, multifunctional cytokine with important biological effects on the vascular endothelium[13]. VEGF mediated blood vessel formation regulates angiogenesis, vasculogenesis, vascular permeability, and in adult angiogenesis, is important in skeletal development and in bone fracture repair[14, 15]. In the context of osteogenesis, VEGF secretion is not only important for stimulation of endothelial cells to undergo proliferation, migration, differentiation and tube formation[16], but also VEGF activities promote bone formation and bone healing by directly influencing the survival, chemotactic migration and activity of osteoblasts[11]. VEGF has three major effects on bone development: (a) induction of angiogenesis in intramembranous or enchondral bone development, (b) chemotactic migration of osteoclastic cells to the hypertrophic cartilage and osteoblastic activation, and (c) direct effects on osteoprogenitor cells by promoting differentiation to osteoblast and increasing mineralization of the regenerated bone[17]. Therefore, the pro-angiogenic cytokine VEGF is a promising biomarker for diagnosis of osteoarthritis and also a prime therapeutic target[11]. There is evidence supporting that high VEGF levels in both plasma and synovial fluids are positively correlated with the radiographic severity of knee osteoarthritis, suggesting increased expression of VEGF occurs in osteoarthritis cartilage, and this may stimulate the growth of blood vessels from subchondral bone into articular cartilage, thereby contributing to osteoarthritis progression[18]. However, other studies found no significant association between VEGF and osteoarthritis[18, 19]. In view of the conflicting results, we performed a meta-analysis to evaluate relationship between increased VEGF expression and the pathogenesis of osteoarthritis.

## Methods

### Literature search and selection criteria

The following electronic databases were searched without language restrictions: MEDLINE (1966 ~ 2013), the Cochrane Library Database (Issue 12, 2013), EMBASE (1980 ~ 2013), CINAHL (1982 ~ 2013), Web of Science (1945 ~ 2013) and the Chinese Biomedical Database (CBM) (1982 ~ 2013). We used the following keywords and MeSH terms in conjunction with a highly sensitive search strategy: [“osteoporosis” or “juvenile osteoporosis” or “age-related osteoporosis” or “age-related bone loss”] and [“osteoarthritis, spine” or “osteoarthritis, knee” or “osteoarthritis” or “coxarthrosis”] and [“vascular endothelial growth factors” or “VEGF” or “vascular endothelial growth factor” or “vascular permeability factor” or “VPF”]. We also conducted a manual search to identify other relevant articles from cross-references.

The following inclusion criteria were employed for the eligibility of studies in our meta-analysis: (1) the study design must be clinical case–control focused on the relationships of VEGF expression levels with pathogenesis of osteoarthritis; (2) all patients met diagnostic criteria for osteoarthritis; (3) the study must provide sufficient information about VEGF expression levels. If the study could not meet the inclusion criteria, it would be excluded. The most recent study or the study with the largest sample size was included when the authors published several studies using the same subjects.

### Data extraction and methodological assessment

Data was extracted from the included studies by two authors using a standardized form. The form used for data extraction documented the most relevant items including language of publication, publication year of article, the first author’s surname, geographical location, design of study, sample size, source of the subjects, VEGF expression levels, source of samples, and detection method.

Methodological quality was evaluated separately by two observers using the Newcastle-Ottawa Scale (NOS) criteria[20]. The NOS criteria included three aspects: (1) subject selection: 0–4; (2) comparability of subject: 0–2; (3) clinical outcome: 0–3. NOS scores ranged from 0 to 9; and a score ≥ 7 indicate a good quality.

### Statistical analysis

Meta-analysis was performed using the STATA statistical software (Version 12.0, Stata Corporation, College Station, TX, USA). Standardized mean difference (SMD) with its corresponding 95% confidence interval (95% CI) was calculated. The *Z* test was used to estimate the statistical significance of pooled SMDs. Heterogeneity among studies were estimated by the Cochran’s *Q*-statistic and *I*^*2*^ tests[21]. If *Q*-test shows a *P* < 0.05 or *I*^*2*^ test exhibits > 50%, which indicates significant heterogeneity, the random-effect model was employed, or else the fixed-effects model was used. We also tested for the source of heterogeneity using subgroup analyses. In order to evaluate the influence of single studies on the overall estimate, a sensitivity analysis was performed. Begger's test and Egger's test were applied to investigate publication bias[22].

## Results

### Study selection and basic characteristics of the included studies

**Figure 1:**
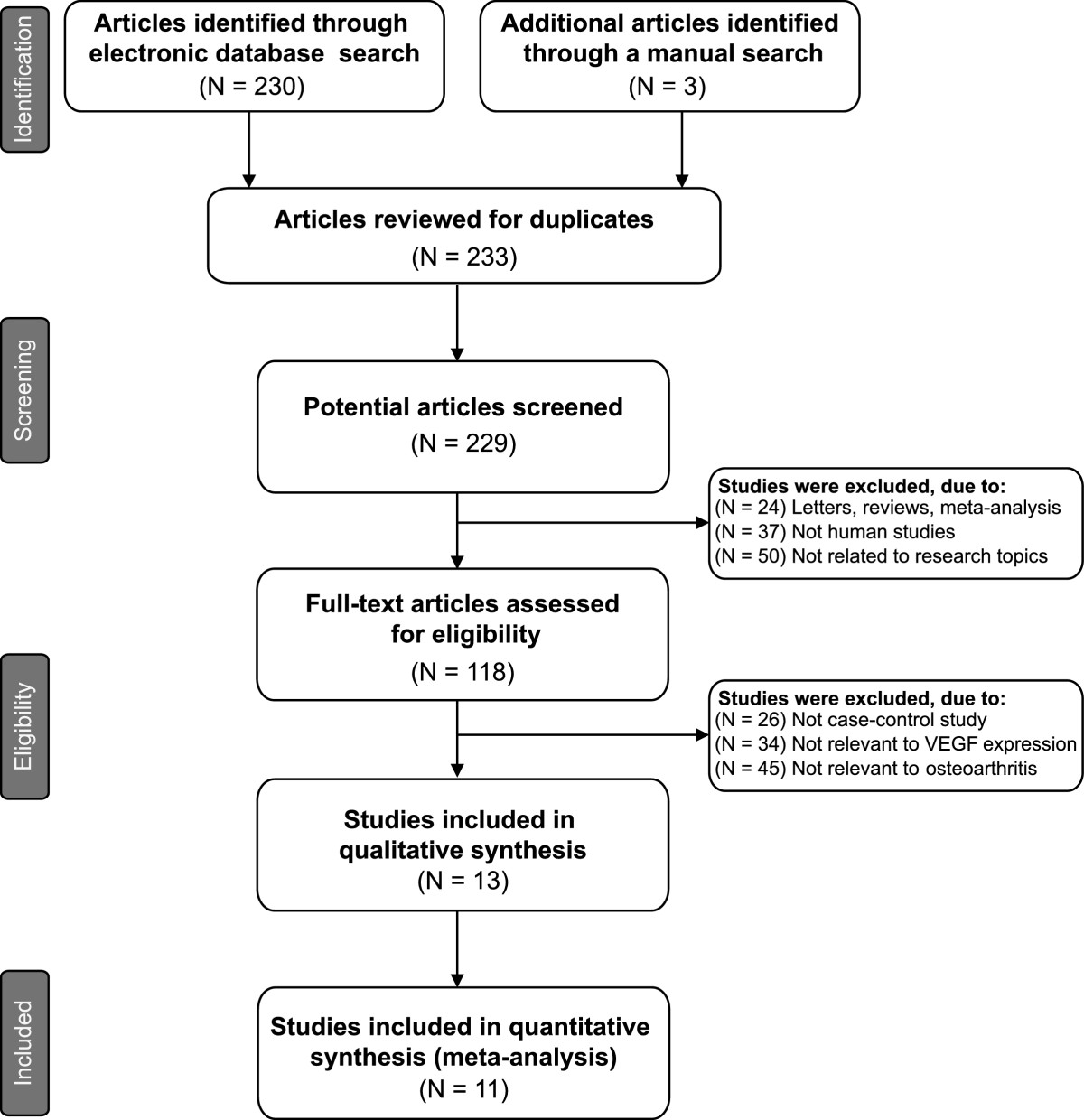
**Flow chart shows study selection procedure.** Eleven case–control studies were included in this meta-analysis.

Our highly sensitive search strategy retrieved 233 articles. After reviewing the titles and abstracts of all the articles, we excluded 111 articles. Next, full texts of the remaining articles were reviewed and 105 articles were further excluded. In addition, 2 studies were excluded due to lack of data integrity (Figure 1). Finally, 11 case–control studies involving a total of 302 osteoarthritis patients and 195 healthy controls met our inclusion criteria for qualitative data analysis[3, 18, 19, 23–30]. These studies were published between 1998 and 2013. Overall, 8 studies were conducted among Asians, and 3 studies among Caucasians. Enzyme-linked immunosorbent assay (ELISA) method was used in all the included studies. Plasma samples were used for VEGF expression levels in 3 studies, 4 studies used cartilaginous tissues, 2 studies used synovial tissues and 2 studies used synovial fluid. NOS scores of all included studies were ≥ 5. We summarized the study characteristics and methodological quality in Table 1.

**Table 1:** **Main characteristics and methodological quality of all eligible studies**

First author	Year	Country	Language	Ethnicity	Gender (M/F)		Age (years)		Method	Protein	Sample source	NOS score
					Case	Control	Case	Control				
Saetan et al.[18]	2013	Thailand	English	Asians	17/63	5/15	69.8 ± 0.9	68.2 ± 1.1	ELISA	VEGF	Plasma	7
Zhu et al.[26]	2012	China	Chinese	Asians	8/10	6/4	65	22	ELISA	VEGF	Synovial fluid	6
Duan et al.[24]	2011	China	English	Asians	8/22	6/4	62 (50 ~ 76)	32	ELISA	VEGF	Synovial tissue	6
Huh et al.[28]	2010	Korea	English	Asians	-	-	74.0 ± 9.0	61.5 ± 15.6	ELISA	VEGF	Cartilaginous tissue	5
Chen et al.[23]	2009	China	Chinese	Asians	11/19	7/1	(52 ~ 75)	(21 ~ 43)	ELISA	VEGF	Synovial tissue	6
Su et al.[25]	2008	China	Chinese	Asians	8/12	7/3	60	45	ELISA	VEGF	Cartilaginous tissue	6
Fay et al.[3]	2006	Germany	English	Caucasians	-	-	-	-	ELISA	VEGF	Synovial fluid	5
Enomoto et al.[27]	2003	Japan	English	Asians	-	-	72.0 ± 8.0	79.0 ± 9.0	ELISA	VEGF	Cartilaginous tissue	5
Pfander et al.[30]	2001	Germany	English	Caucasians	-	-	-	-	ELISA	VEGF	Cartilaginous tissue	5
Lee et al.[29]	2001	Korea	English	Asians	4/45	-	59.6 ± 1.1	-	ELISA	VEGF	Plasma	5
Ballara et al.[19]	2001	Germany	English	Caucasians	15/17	11/20	55 (50 ~ 65)	49 (38 ~ 55)	ELISA	VEGF	Plasma	6

M: male; F: female; NOSF: Newcastle-Ottawa Scale; VEGF: vascular endothelial growth factor; ELISA: enzyme-linked immunosorbent assay.

### Quantitative data synthesis

**Figure 2:**
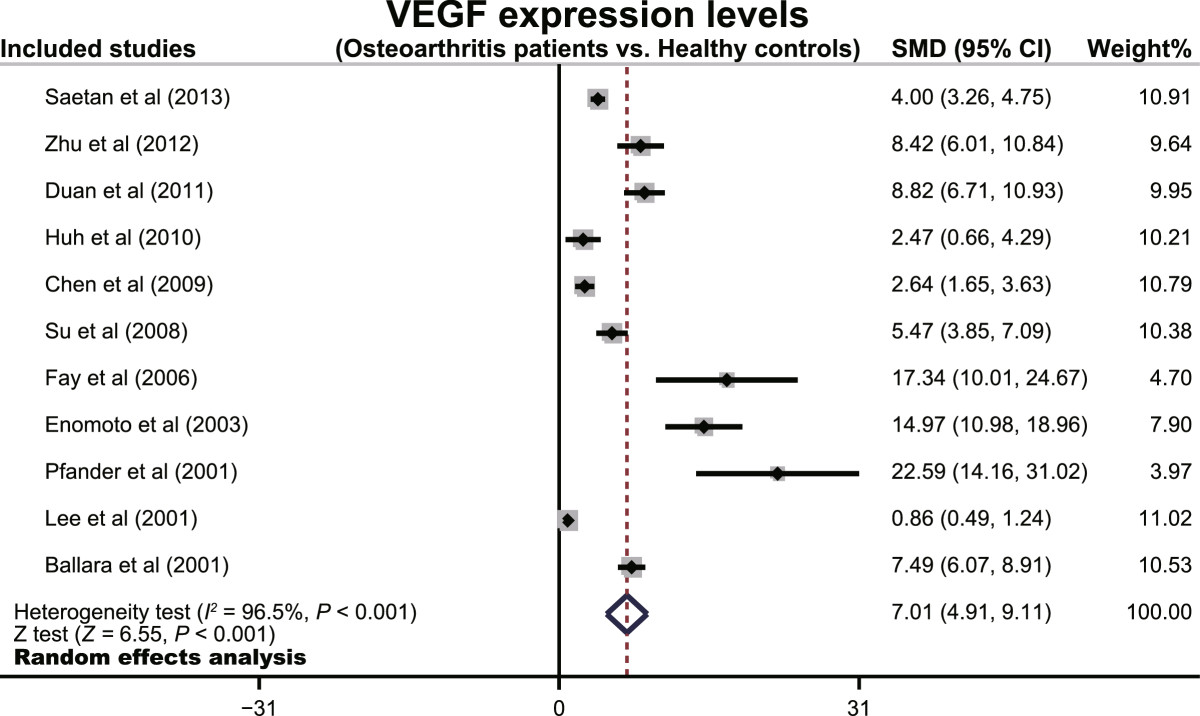
**Forest plots for the relationships between VEGF expression levels and the pathogenesis of osteoarthritis.**

**Figure 3:**
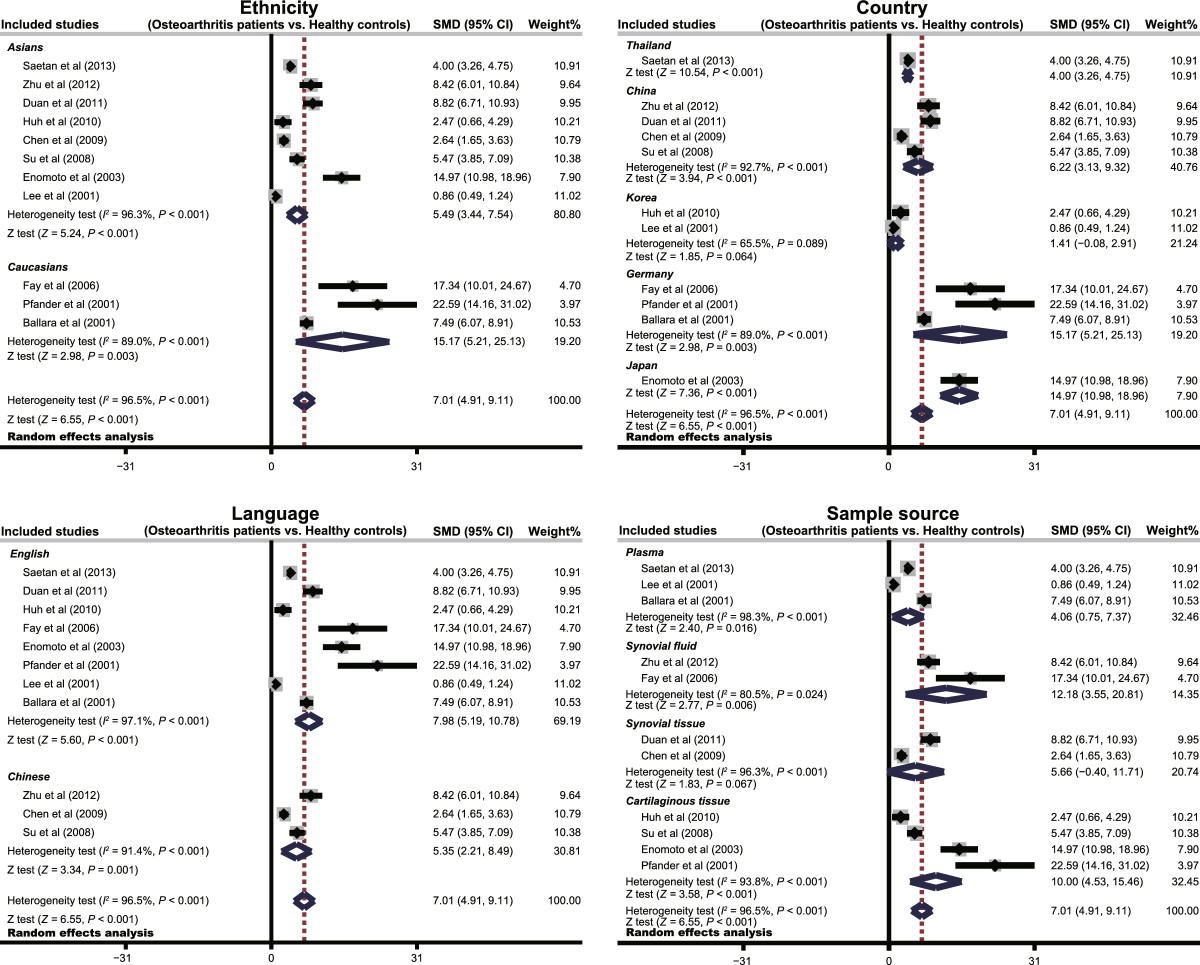
**Subgroup analyses by ethnicity, country, language and sample source of the relationships between VEGF expression levels and the pathogenesis of osteoarthritis.**

**Figure 4:**
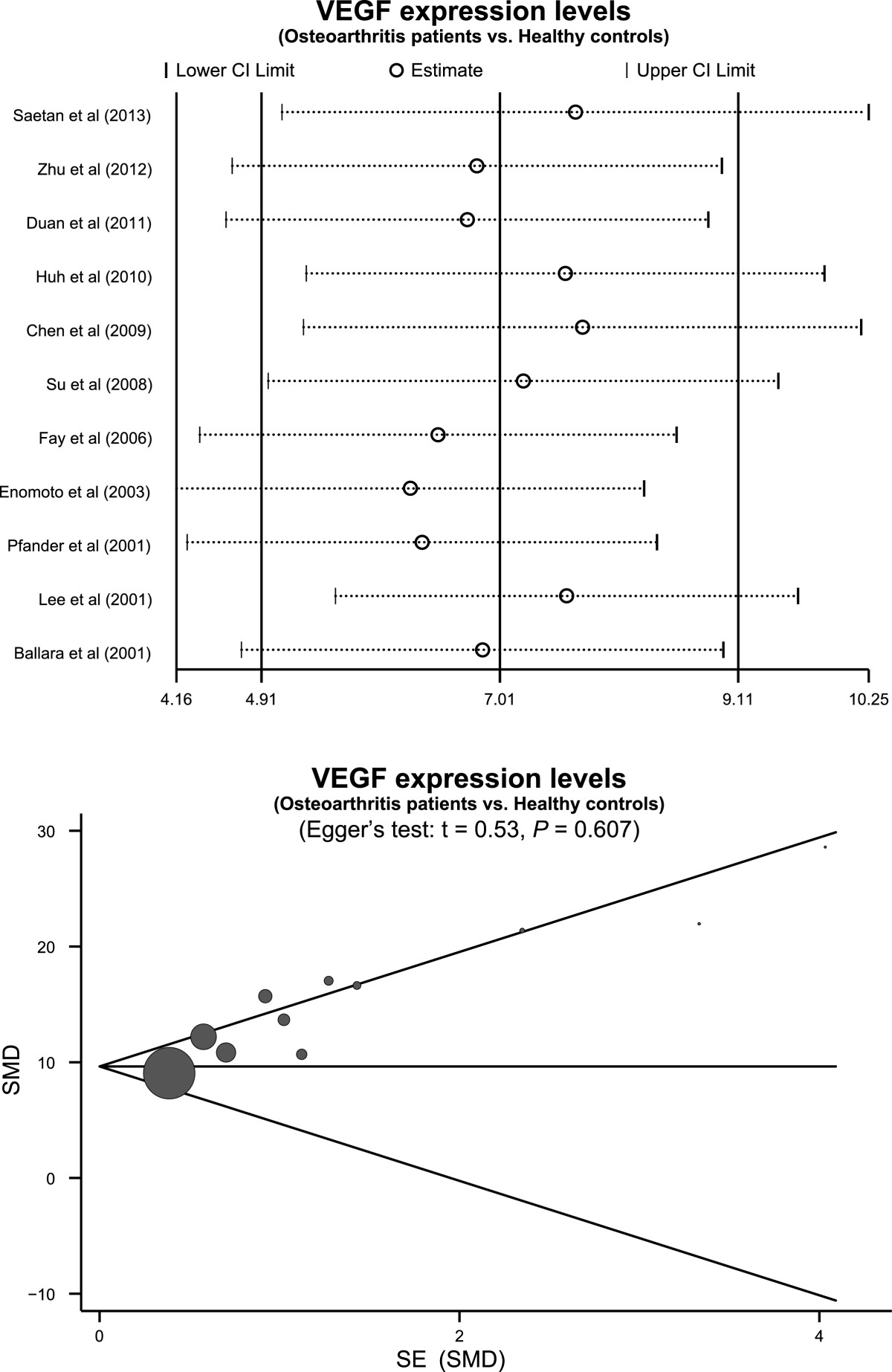
**Sensitivity analysis and publication bias evaluation of the summary estimate coefficients on the relationships between VEGF expression levels and the pathogenesis of osteoarthritis.**

The random effects model was employed since significant heterogeneity existed between studies (*I*^*2*^ = 96.5%, *P* < 0.001). The results of our meta-analysis revealed that VEGF expression levels in osteoarthritis patients were significantly higher than healthy controls (SMD = 7.01, 95% CI: 4.91 ~ 9.11, *P* < 0.001) (Figure 2). A subgroup analysis based on ethnicity, revealed that both Asian and Caucasian osteoarthritis patients had higher levels of VEGF expression than healthy controls(Asians: SMD = 5.49, 95% CI: 3.44 ~ 7.54, *P* < 0.001; Caucasians: SMD = 15.17, 95% CI: 5.21 ~ 25.13, *P* = 0.003; respectively) (Figure 3). We performed other subgroup analyses based on country, language and sample source. The results also suggested that osteoarthritis patients had higher levels of VEGF expression than healthy controls in all the subgroups (all *P* > 0.05). Sensitivity analysis suggested that no single study could influence the pooled ORs (Figure 4). Funnel plots demonstrated no evidence of asymmetry. Egger’s test also did not display statistical evidence for publication bias (*t* = 0.53, *P* = 0.607) (Figure 4).

## Discussion

We investigated the relationship between steady-state VEGF levels and osteoarthritis. Our meta-analysis showed a positive association of VEGF expression levels with osteoarthritis, implying that VEGF may play an important role in osteoarthritis progression. Environmental, biomechanical and biological factors, such as growth factors, play a substantial role in the development of osteoarthritis[31]. Although VEGF’s role is suspected, the precise mechanism by which VEGF levels influence osteoarthritis remains largely unknown. We hypothesize that increased local production of VEGF plays a pivotal role in endochondral ossification by coupling angiogenesis with hypertrophic cartilage remodelling and endochondral bone formation, through acting on osteoblasts[27]. VEGF expression is also upregulated in inflammatory arthritis, suggesting VEGF may have specific roles in angiogenesis and inflammation, which are closely related processes in osteoarthritis[32]. Our meta-analysis finding is in accordance with a previous study that demonstrated increased VEGF expression by chondrocytes in osteoarthritic articular cartilage, suggesting chondrocyte involvement in articular cartilage destruction and development of osteoarthritis[3]. Consistent with the argument for an important role of VEGF in osteoarthritis, Gallelli et al. showed that ibuprofen, diclofenac and celecoxib decrease VEGF levels in the synovial fluid of the osteoarthritic joint, with a concomitant improvement in joint pain as well as joint function in osteoarthritis patients[33]. Thus, VEGF could serve as a promising biomarker to assessdisease severity in osteoarthritis, and could be the basis for targeted clinical treatments.

In order to understand the correlations between VEGF expression levels and osteoarthritis, we performed subgroup analyses based on ethnicity, country, language and sample source. Our meta-analysis results conclusively showed that the expression levels of VEGF were significantly higher in osteoarthritis patients than healthy controls in all these subgroups, indicating the high value of VEGF as a reliable disease biomarker VEGF plays an important role in bone formation, mineralization, and remodeling, by acting directly on the differentiation and survival of osteoblasts[34]. Consistent with this, inhibition of VEGF mediated signaling by neutralization of VEGF receptor decreased angiogenesis, bone formation and callus mineralization in experimental animal models, suggesting the significant potential of the VEGF pathway in addressing bone diseases[9, 11]. However, strategies that involve inhibition of VEGF pathways must also be approached with caution due to the central role played by VEGF in many normal cellular processes. For example, by promoting endothelial cells proliferation, VEGF enhances vascular permeability, and thereby contributes to blood vessel formation in the bone, which is an important step in the healing of osteoporotic bone[35, 36]. Also, interestingly, decreased secretion of VEGF, causes increased intracellular accumulation of VEGF, and is important in osteoblast and adipocyte differentiation choice, and an imbalance could potentially contribute to the severity and progression of osteoarthritis[37]. Martinez et al. observed an age-related decrease in secreted VEGF in osteoblasts, implicating VEGF as the modulating factor for bone remodeling in patients with osteoarthritis[38]. Taken together, we speculate that VEGF plays an important role in normal bone growth and in the pathogenesis of osteoarthritis, and is a promising biomarker for determining the disease severity in osteoarthritis.

The current meta-analysis also had several limitations that should be mentioned. First, our results lack sufficient statistical power to assess the correlations of VEGF expression levels with the pathogenesis of osteoarthritis due to the small number of studies. The small number of studies may restrict general applications of our findings, and consequently our meta-analysis should be regarded as preliminary. Second, since we only included studies written in English and Chinese, a potential selection bias cannot be totally excluded. Third, we could not investigate the detailed evaluation of VEGF in the pathogenesis of osteoarthritis due to the lack of original data in the included studies.

## Conclusion

In conclusion, our meta-analysis revealed that VEGF expression levels strongly correlate with the pathogenesis of osteoarthritis. Thus, we believe VEGF should be considered as a promising biomarker to assess disease severity in osteoarthritis, and could be the basis for targeted treatments in the future. However, due to the limitations in our current study, larger sample size and more detailed information are required to conclusively prove our findings in this meta-analysis.

## Supporting Information

Our manuscript reporting adheres to MOOSE guidelines for reporting meta-analysis observational studies in epidemiology.The supporting MOOSE Checklist is available as supplementary information; see Additional file1.

## Electronic supplementary material

Additional file 1:MOOSE guidelines for reporting meta-analysis observational studies in epidemiology.(DOC 71 KB)

Below are the links to the authors’ original submitted files for images.

Authors’ original file for figure 1

Authors’ original file for figure 2

Authors’ original file for figure 3

Authors’ original file for figure 4

Authors’ original file for figure 5

Authors’ original file for figure 6

## Abbreviations

VEGF: Vascular endothelial growth factor
CBM: Chinese Biomedical Database
SMD: Standardized mean difference
95% CI: 95% confidence interval
ECM: Extracellular matrix
ELISA: Enzyme-linked immunosorbent assay.
