# Supplementary material for: Elevated VEGF levels contribute to the pathogenesis of osteoarthritis
Source: BMC Musculoskelet Disord. 2014 Dec 17;15:437. doi: 10.1186/1471-2474-15-437 (PMC4391471; doi:10.1186/1471-2474-15-437)
Supplement: Supplementary file 1 — Additional file 1:MOOSE guidelines for reporting meta-analysis observational studies in epidemiology.(DOC 71 KB) [file 12891_2014_2444_MOESM1_ESM.doc]

**MOOSE Checklist for Meta-analyses of Observational Studies**

| **Item No** | **Recommendation** | **Reported on Page No** |
| --- | --- | --- |
| Reporting of background should include | | |
| 1 | Problem definition | Page 5 |
| 2 | Hypothesis statement | Page 5 |
| 3 | Description of study outcome(s) | Page 3 |
| 4 | Type of exposure or intervention used | Page 3 |
| 5 | Type of study designs used | Page 3 |
| 6 | Study population | Page 3 |
| Reporting of search strategy should include | | |
| 7 | Qualifications of searchers (eg, librarians and investigators) | Page 5 |
| 8 | Search strategy, including time period included in the synthesis and key words | Page 5-6 |
| 9 | Effort to include all available studies, including contact with authors | Page 6 |
| 10 | Databases and registries searched | Page 5 |
| 11 | Search software used, name and version, including special features used (eg, explosion) | Page 6 |
| 12 | Use of hand searching (eg, reference lists of obtained articles) | Page 6 |
| 13 | List of citations located and those excluded, including justification | Page 6 |
| 14 | Method of addressing articles published in languages other than English | Page 6 |
| 15 | Method of handling abstracts and unpublished studies | Page 6 |
| 16 | Description of any contact with authors | Page 6 |
| Reporting of methods should include | | |
| 17 | Description of relevance or appropriateness of studies assembled for assessing the hypothesis to be tested | Page 6 |
| 18 | Rationale for the selection and coding of data (eg, sound clinical principles or convenience) | Page 7 |
| 19 | Documentation of how data were classified and coded (eg, multiple raters, blinding and interrater reliability) | Page 7 |
| 20 | Assessment of confounding (eg, comparability of cases and controls in studies where appropriate) | Page 7 |
| 21 | Assessment of study quality, including blinding of quality assessors, stratification or regression on possible predictors of study results | Page 7 |
| 22 | Assessment of heterogeneity | Page 7 |
| 23 | Description of statistical methods (eg, complete description of fixed or random effects models, justification of whether the chosen models account for predictors of study results, dose-response models, or cumulative meta-analysis) in sufficient detail to be replicated | Page 7 |
| 24 | Provision of appropriate tables and graphics | Page 7 |
| Reporting of results should include | | |
| 25 | Graphic summarizing individual study estimates and overall estimate | Page 8 |
| 26 | Table giving descriptive information for each study included | Page 7 |
| 27 | Results of sensitivity testing (eg, subgroup analysis) | Page 8 |
| 28 | Indication of statistical uncertainty of findings | Page 8 |

| **Item No** | **Recommendation** | **Reported on Page No** |
| --- | --- | --- |
| Reporting of discussion should include | | |
| 29 | Quantitative assessment of bias (eg, publication bias) | Page 7 |
| 30 | Justification for exclusion (eg, exclusion of non-English language citations) | Page 7 |
| 31 | Assessment of quality of included studies | Page 10 |
| Reporting of conclusions should include | | |
| 32 | Consideration of alternative explanations for observed results | Page 8,9 |
| 33 | Generalization of the conclusions (ie, appropriate for the data presented and within the domain of the literature review) | Page 10 |
| 34 | Guidelines for future research | Page 10 |
| 35 | Disclosure of funding source | Page 11 |

Transcribed from the original paper within the NEUROSURGERY® Editorial Office, Atlanta, GA, United Sates. August 2012.
