# Supplementary figures and images for: Elevated VEGF levels contribute to the pathogenesis of osteoarthritis
Source: BMC Musculoskelet Disord. 2014 Dec 17;15:437. doi: 10.1186/1471-2474-15-437 (PMC4391471; doi:10.1186/1471-2474-15-437)

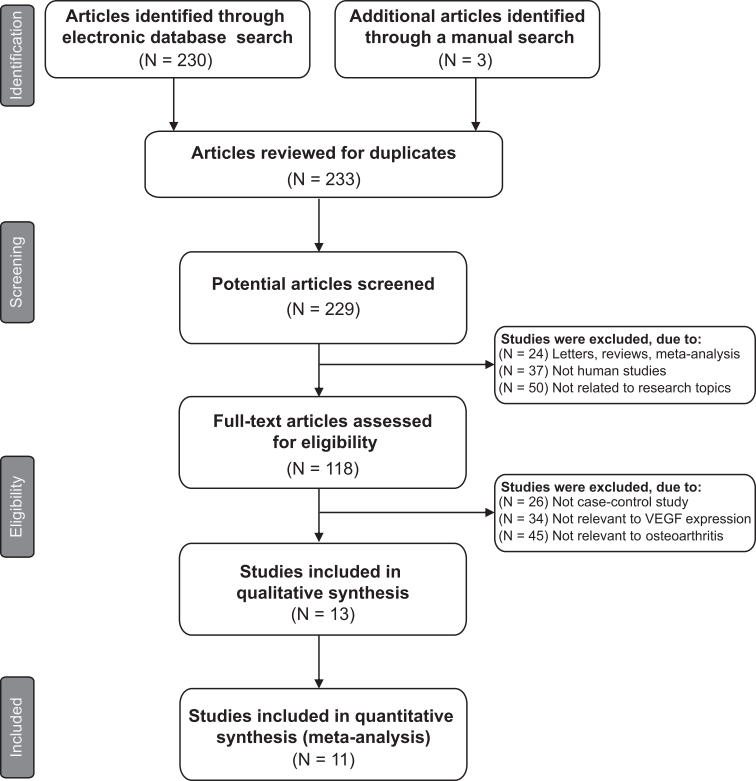

Supplement: Supplementary file 2 — Authors’ original file for figure 1 [file 12891_2014_2444_MOESM2_ESM.pdf]

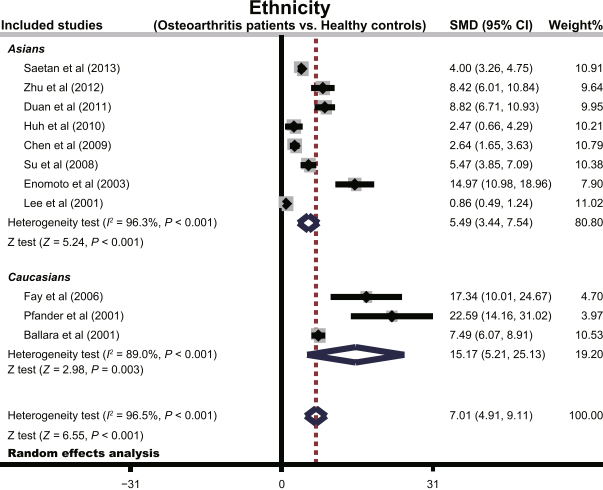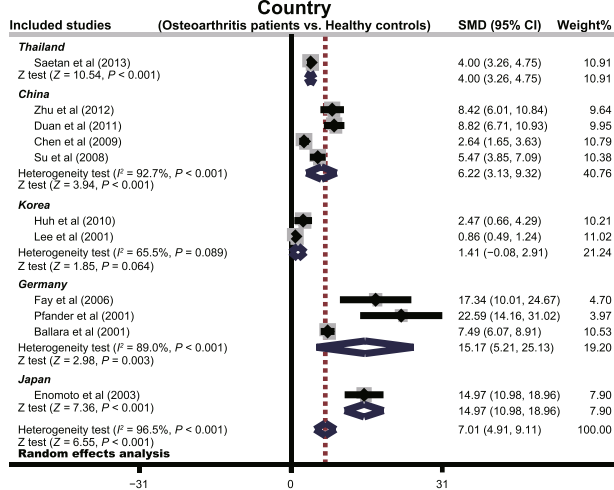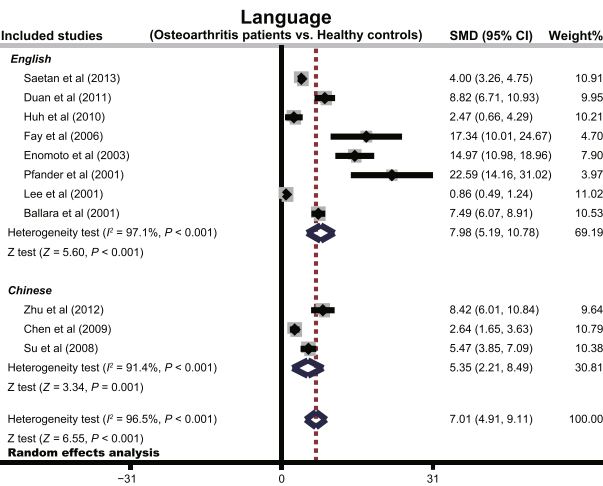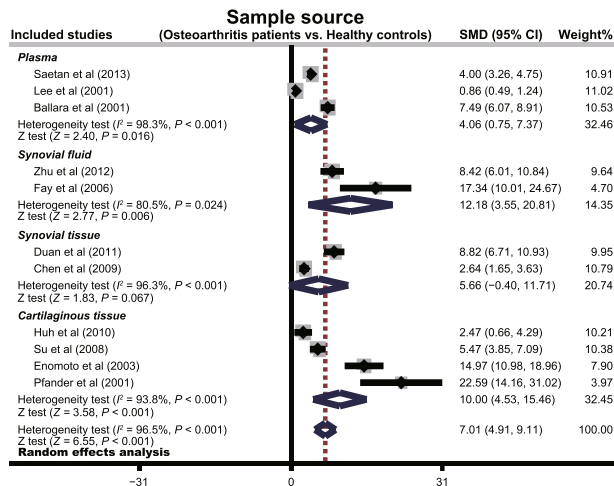

Supplement: Supplementary file 4 — Authors’ original file for figure 3 [file 12891_2014_2444_MOESM4_ESM.pdf]
