# Supplementary material for: Elevated VEGF levels contribute to the pathogenesis of osteoarthritis
Source: BMC Musculoskelet Disord. 2014 Dec 17;15:437. doi: 10.1186/1471-2474-15-437 (PMC4391471; doi:10.1186/1471-2474-15-437)
Supplement: Supplementary file 3 — Authors’ original file for figure 2 [file 12891_2014_2444_MOESM3_ESM.pdf]

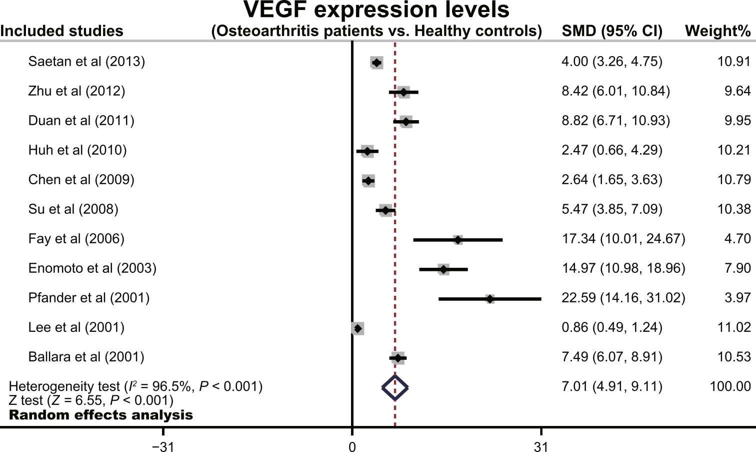

VEGF expression levels
Included studies
(Osteoarthritis patients vs. Healthy controls)
SMD (95% CI)
Weight%
Saetan et al (2013)
4.00 (3.26, 4.75)
10.91
Zhu et al (2012)
8.42 (6.01, 10.84)
9.64
Duan et al (2011)
8.82 (6.71, 10.93)
9.95
Huh et al (2010)
2.47 (0.66, 4.29)
10.21
Chen et al (2009)
2.64 (1.65, 3.63)
10.79
Su et al (2008)
5.47 (3.85, 7.09)
10.38
Fay et al (2006)
17.34 (10.01, 24.67)
4.70
Enomoto et al (2003)
14.97 (10.98, 18.96)
7.90
Pfander et al (2001)
22.59 (14.16, 31.02)
3.97
Lee et al (2001)
0.86 (0.49, 1.24)
11.02
Ballara et al (2001)
7.49 (6.07, 8.91)
10.53
Heterogeneity test (I^2 = 96.5%, P < 0.001)
7.01 (4.91, 9.11)
100.00
Z test (Z = 6.55, P < 0.001)
Random effects analysis
−31
0
31
