# Supplementary material for: Elevated VEGF levels contribute to the pathogenesis of osteoarthritis
Source: BMC Musculoskelet Disord. 2014 Dec 17;15:437. doi: 10.1186/1471-2474-15-437 (PMC4391471; doi:10.1186/1471-2474-15-437)
Supplement: Supplementary file 7 — Authors’ original file for figure 6 [file 12891_2014_2444_MOESM7_ESM.doc]

**Table 1** Main characteristics and methodological quality of all eligible studies

| First author | Year | Country | Language | Ethnicity | Gender (M/F) | | Age (years) | | Method | Protein | Sample source | NOS score |
| --- | --- | --- | --- | --- | --- | --- | --- | --- | --- | --- | --- | --- |
|  |  |  |  |  |  |  |  |  |  |  |  |  |
|  |  |  |  |  | Case | Control | Case | Control |  |  |  |  |
| Saetan et al [18] | 2013 | Thailand | English | Asians | 17/63 | 5/15 | 69.8 ± 0.9 | 68.2 ± 1.1 | ELISA | VEGF | Plasma | 7 |
| Zhu et al [26] | 2012 | China | Chinese | Asians | 8/10 | 6/4 | 65 | 22 | ELISA | VEGF | Synovial fluid | 6 |
| Duan et al [24] | 2011 | China | English | Asians | 8/22 | 6/4 | 62 (50~76) | 32 | ELISA | VEGF | Synovial tissue | 6 |
| Huh et al [28] | 2010 | Korea | English | Asians | - | - | 74.0 ± 9.0 | 61.5 ± 15.6 | ELISA | VEGF | Cartilaginous tissue | 5 |
| Chen et al [23] | 2009 | China | Chinese | Asians | 11/19 | 7/1 | (52~75) | (21~43) | ELISA | VEGF | Synovial tissue | 6 |
| Su et al [25] | 2008 | China | Chinese | Asians | 8/12 | 7/3 | 60 | 45 | ELISA | VEGF | Cartilaginous tissue | 6 |
| Fay et al [3] | 2006 | Germany | English | Caucasians | - | - | - | - | ELISA | VEGF | Synovial fluid | 5 |
| Enomoto et al [27] | 2003 | Japan | English | Asians | - | - | 72.0 ± 8.0 | 79.0 ± 9.0 | ELISA | VEGF | Cartilaginous tissue | 5 |
| Pfander et al [30] | 2001 | Germany | English | Caucasians | - | - | - | - | ELISA | VEGF | Cartilaginous tissue | 5 |
| Lee et al [29] | 2001 | Korea | English | Asians | 4/45 | - | 59.6 ± 1.1 | - | ELISA | VEGF | Plasma | 5 |
| Ballara et al [19] | 2001 | Germany | English | Caucasians | 15/17 | 11/20 | 55 (50~65) | 49 (38~55) | ELISA | VEGF | Plasma | 6 |

*M* male, *F*  female, *NOS* Newcastle-Ottawa Scale, *VEGF* vascular endothelial growth factor, *ELISA* enzyme-linked immunosorbent assay
